# miR-96-5p Induces Orbital Fibroblasts Differentiation by Targeting Smad7 and Promotes the Development of Thyroid-Associated Ophthalmopathy

**DOI:** 10.1155/2022/8550307

**Published:** 2022-02-27

**Authors:** Jianshu Kang, Yunqin Li, Yue Zou, Zhijian Zhao, Linan Jiao, Hong Zhang

**Affiliations:** Department of Ophthalmology, The Affiliated Hospital of Yunnan University, Kunming 650021, China

## Abstract

**Background:**

Recent evidence shows that adipogenic differentiation of orbital fibroblasts (OFs) promotes the development of thyroid-associated ophthalmopathy (TAO), an organ-specific immune disease. Furthermore, miR-96-5p has been linked to adipogenic differentiation of C2C12 myoblasts and is significantly correlated with the severity of TAO. The purpose of this study is to look into the role of miR-96-5p in the adipogenesis of OFs with TAO.

**Methods:**

The orbital tissues from TAO patients and non-TAO participants were collected, and primary OFs were isolated and cultured for further analysis. miR-96-5p expression was examined using qRT-PCR. The adipogenic differentiation of OFs was then studied.

**Results:**

Orbital fibroblasts isolated from adipose tissues of TAO patients (t-OFs) demonstrated greater adipogenic differentiation ability than OFs isolated from adipose tissues of non-TAO participants. miR-96-5p was found to be overexpressed in the orbital tissues of TAO patients and t-OFs. Further research revealed that miR-96-5p, by targeting Smad7, could exacerbate PPAR-*γ*/C/EBP*α* signaling-induced adipogenic differentiation of t-OFs. However, inhibiting miR-96-5p could block t-OFs adipogenic differentiation-mediated adipogenesis via Smad7/PPAR-*γ*/C/EBP*α*.

**Conclusions:**

miR-96-5p plays a critical regulatory role in the development of TAO by targeting Smad7 and promoting adipogenic differentiation of OFs.

## 1. Introduction

Thyroid-associated ophthalmopathy (TAO), also known as Graves' ophthalmopathy, is an organ-specific immune disease marked by increased adipose/connective tissue volume and the potential for blindness. It is one of the most common ophthalmic diseases in clinical practice, with TAO having the highest prevalence among orbital diseases [1, 2]. TAO, on the other hand, is a common extrathyroid manifestation and has in recent years been linked to hypothyroidism, subacute thyroiditis, and thyroid cancer [3]. At present, TAO research currently focuses primarily on immunology [4], pathology [5], genetic background [6, 7], and environmental factors [8, 9], but the pathogenesis and pathogenesis of TAO remain unknown [10].

Emerging evidence shows that TAO symptoms are caused by inflammation of orbital connective tissue. The infiltration of inflammatory cells, the accumulation of extracellular matrix proteins, the proliferation of fibroblasts, and the increasing of orbital adipose tissue causes the expansion of orbital connective tissue This results in orbital tissue fibrosis, orbital tissue remodeling, and destruction of adjacent eyeball structure and function, promoting the development of TAO [11, 12]. Orbital fibroblast (OFs) are thought to be important immune targets and effector cells in the development of TAO [13, 14]. Current research evidence indicates that fibrosis and inflammatory factor secretions mediated by OFs play a role in the development of TAO [15]. Furthermore, adipogenic differentiation of OFs is important in the increase of orbital adipose tissue in TAO [16, 17], and adipogenesis mediated by adipogenic differentiation of activated OFs may result in the orbital protrusion in TAO patients [18]. However, the molecular mechanism of adipogenic differentiation of orbital fibroblasts during TAO needs to be investigated further.

MicroRNAs (miRNAs) are endogenous single-stranded noncoding small mRNAs. Approximately, 90% of miRNAs are expressed differently in different parts of the human eye, and each miRNA can play a unique role in eye tissues [19]. Moreover, an increasing body of evidence suggests that miRNAs are critical in regulating adipogenesis [20]. miR-96-5p has been linked to adipogenic differentiation of C2C12 myoblasts [21]. A previous study found that miR-96-5p was highly expressed in thyroid tissues of patients with autoimmune thyroid disease and was significantly positively correlated with TAO severity [22]. These findings suggested that high expression of miR-96-5p is associated with higher severity of diseases in patients with Graves' orbitopathy (GO), including active eye disease, goiter, high antibody titer, and/or higher recurrence rate. Previous research on miR-96-5p has mostly focused on its role in tumor occurrence and development [23–25]. Additionally, one study found miR-96-5p to be a potential biomarker for multisystem atrophy, Parkinson's disease, and gestational diabetes mellitus [26]. However, the role of miR-96-5p in the adipogenic differentiation of OFs in TAO is still unknown.

Smad7 is a key negative regulator in the TGF-*β* signal transduction pathway, and its role in osteogenic differentiation has been well documented [27, 28]. Recent evidence has shown a role for Smad7 in adipogenesis [29, 30]. While peroxisome proliferator-activated receptor-*γ* (PPAR-*γ*) signal transduction has been identified as the primary inducer of adipogenesis [31, 32], studies have revealed interactions between TGF-*β* signaling and PPAR-*γ* signaling [33]. Studies have shown that the Smad signal plays an important role in periorbital fibrosis in TAO [34–36], and Smad7 is predicted to be one of the target genes of miR-96-5p (StarBase, URL: http://starbase.sysu.edu.cn/agoClipRNA.php?source=mRNA). The present study, however, explores whether miR-96-5p plays a role in adipogenic differentiation of OFs during TAO progression via Smad7/PPAR-*γ* signaling.

The present study demonstrated that Smad7 is a target gene of miR-96-5p, and miR-96-5p is noticeably overexpressed in TAO orbital adipose/connective tissues and OFs. The findings provide evidence that miR-96-5p promotes adipogenic differentiation of OFs by targeting Smad7 and activating PPAR-*γ* signaling, thereby promoting TAO adipogenesis.

## 2. Materials and Methods

### 2.1. Sample Collection and Cell Culture

Orbital adipose tissue was obtained from 15 TAO patients and 10 non-TAO subjects as described in a previous study [16]. All participants provided informed written consent. The Institutional Review Board (IRB) of the Affiliated Hospital of Yunnan University approved this study (approval no. 2019173). The following were the inclusion criteria for TAO patients: (1) patients had orbital decompression for proptosis correction; (2) patients were euthyroid and had inactive TAO status at the time of surgery; (3) patients had not been treated with steroids or radiation therapy for at least 3 months. For non-TAO subjects: (1) age- and sex-matched to TAO subjects; (2) no thyroid or other inflammatory diseases were present; (3) control subjects underwent cosmetic upper and lower blepharoplasty.

Cultures of primary orbital fibroblasts (OFs) were grown following previously described methods [37]. Briefly, the tissue blocks were cut into pieces and placed in a DMEM medium containing 20% FBS, 100 U/mL penicillin, and 20 *μ*g/mL gentamicin from Hyclone Laboratories (Logan, UT). The explants were cultured until fibroblasts formed a monolayer and grew out of the explants. The monolayer cells were then mildly digested with trypsin/EDTA, and subcultured in a DMEM medium containing 10% FBS in a humidified 5% CO_2_ incubator at 37°C. Following cell sorting and flow cytometry detection of the surface antigen of the obtained cells, cells of the third to seventh generations in good condition were used for subsequent cell experiments. In comparison to OFs obtained from orbital adipose tissues of non-TAO subjects (n-OFs), the orbital adipose tissues obtained from TAO patients were referred to as t-OFs in the following study.

### 2.2. Cell Transfection

Orbital fibroblasts were transfected with the miR-96-5p inhibitor, siRNA of Smad7 (siSmad7), and each control following the manufacturer's protocols. The miR-96-5p inhibitor and siSmad7 were obtained from Guangzhou RiboBio Biotechnology Co., Ltd. (Guangzhou; China). Cells were transfected with 50 nM of miR-96-5p inhibitor or siSmad7 using commercial Lipofectamine® 2000 transfection reagent (Invitrogen; Thermo Fisher Scientific, Inc.; USA) following the manufacturer's instructions and recent study [38]. Briefly, the cells were incubated with 50 nM of miR-96-5p inhibitor or siSmad7 for 48 to 72 hours and maintained under normal growth conditions. At 72 hours, qRT-PCR was used to confirm the efficiency of transfection of miR-96-5p inhibitor and siSmad7.

### 2.3. Dual-Luciferase Reporter Assay

The binding sites between miR-96-5p and Smad7 were predicted with StarBase (URL: http://starbase.sysu.edu.cn/agoClipRNA.php?source=mRNA). Luciferase vectors containing the 3'UTR of human Smad7 with the miR-96-5p binding sites and mutant miR-96-5p binding sites were purchased from Shanghai GenePharma Co., Ltd. The vectors were cotransfected into 293T cells with miR-96-5p mimics by Lipofectamine® 2000 transfection reagent (Invitrogen; Thermo Fisher Scientific, Inc.; USA). The luciferase reporter activity was measured after 48 h using a Dual-Luciferase® eporter Assay System (Promega Corporation). This assay was performed according to the previous description [38].

### 2.4. Quantitative Real-Time Polymerase Chain Reaction (qRT-PCR)

The expression level of RNA was determined by qRT-PCR according to the previous study [38]. In detail, total RNA was extracted from tissues and cells using Trizol reagent (Invitrogen; Thermo Fisher Scientific, Inc.; USA) according to the manufacturer's instructions to detect the expression levels of miR-96-5p in orbital tissues and OFs. miRNA qRT-PCR was performed using a TaqMan™ MicroRNA Reverse Transcription kit (Applied Biosystems; Thermo Fisher Scientific, Inc.; USA) and a TaqMan Universal PCR Master Mix (Applied Biosystems; Thermo Fisher Scientific, Inc.; USA). The 2^−ΔΔCq^ method [39] was used to present the relative expressions of miRNA as fold changes, and U6 was used to normalize the miRNA level. The primer sequences used were as follows: human U6, forward: 5′-CTCGCTTCGGCAGCACATATACT-3′and reverse: 5′-ACGCTTCACGAATTTGCGTGTC-3′; human miR-96-5p, forward: 5′- CAGTCGTTTTTACACGATCAC-3′ and reverse: 3′- GGTCCAGTTTTTTTTTTTTTTTAAACC-5′.

### 2.5. Western Blotting

Orbital tissues and fibroblast cells were harvested and lysed in RIPA buffer containing protease inhibitors (Invitrogen; USA), and protein concentrations were determined using the Pierce BCA assay (Invitrogen; USA), following the manufacturer's protocols. The proteins in lysates (40 *μ*g of each sample) were then separated by SDS-PAGE and transferred to PVDF membranes. The membranes were blocked with 5% nonfat milk for 1 h at room temperature and subsequently incubated with primary antibodies overnight at 4°C. Expression levels of the proteins of interest were analyzed using primary antibodies against Smad7, PPAR-*γ*, C/EBP*α*, adiponectin, and FABP-4 purchased from Abcam, UK, at a dilution of 1 : 1000. Membranes were rinsed three times with 1X Tris-buffered saline containing 0.5% Tween-20 (TBST) and then incubated for 1 h with anti-rabbit IgG (1 : 2000, Abcam, UK) horseradish peroxidase-conjugated secondary antibody. Membranes were rinsed three times with TBST and examined with an ECL kit (Bio-Rad Laboratories, Inc.). The protein bands were quantified using the ImageJ software (version 1.52a; National Institutes of Health), and GAPDH (1 : 1000, Abcam, UK) was used as a loading control. Each experiment was performed in triplicate.

### 2.6. Oil Red O Staining

Briefly, cells were fixed in 4% paraformaldehyde (PFA) for 15 min and then washed three times with PBS for 5 min each. After that, cells were incubated for 10–15 min with oil red O working solution (oil red: distilled water = 3 : 2) at room temperature. Following that, 60% isopropanol was used to separate the samples for 30 s before washing with distilled water for 1 min. Finally, the filter paper was used to absorb the surrounding water, and the cells were sealed with glycerin gelatin. As a result, the lipid droplets stained orange-red to bright red.

### 2.7. Statistical Analyses

All experiments were performed at least three times independently, with at least three cell cultures harvested from different individuals. The results are presented in the form of the mean ± standard deviation. Differences between groups were assessed by Students' *t*-tests and one-way ANOVA or two-way ANOVA followed by Bonfferroni's multiple comparisons test. In all analyses, *P* <  0.05 denoted statistical significance.

## 3. Results

### 3.1. miR-96-5p Is Highly Expressed in Orbital Tissue and Orbital Fibroblasts of TAO Patients

**Figure 1:**
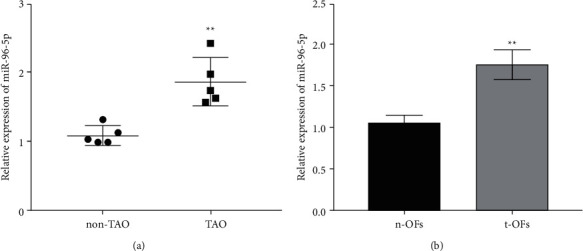
The expression of miR-96-5p in orbital tissues and orbital fibroblasts from non-TAO and TAO participants. (a) The relative expression of miR-96-5p in orbital tissues measured by qRT-PCR. (b) The relative expression levels of miR-96-5p in OFs separated from adipose tissues with or without TAO. ^*∗∗*^*P* <  0.01 vs non-TAO or n-OFs group by two-tailed Students' *t*-test.

The expression of miR-96-5p in orbital tissues and OFs from TAO patients was investigated in qRT-PCR assays. The results showed that miR-96-5p was significantly upregulated in the orbital tissues of TAO patients compared to non-TAO participants (Figure 1(a)). Furthermore, the expression of miR-96-5p in t-OFs was noticeably higher than that in n-OFs (Figure 1(b)), whereas the relative expression level of miR-96-5p in t-OFs was nearly 1.5 times that of n-OFs. These findings suggested that miR-96-5p upregulation may play an important role in the progression of TAO and influence the bioactivity of t-OFs.

### 3.2. t-OFs Show a Higher Ability of Adipogenesis

**Figure 2:**
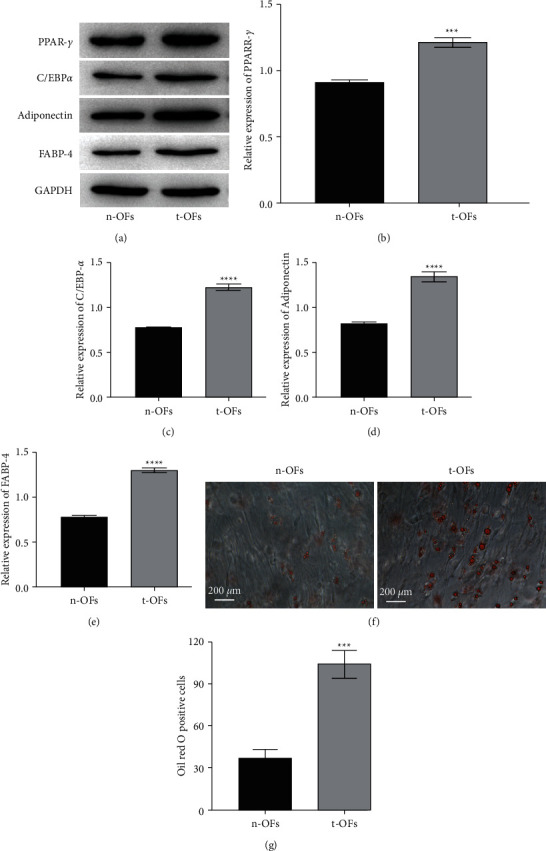
The adipogenesis ability of OFs from non-TAO and TAO participants. (a) The representative bands of western blotting for PPAR-*γ*, C/EBP*α*, adiponectin, and FABP-4. (b–e) The relative expression levels of PPAR-*γ*, C/EBP*α*, adiponectin, and FABP-4 in n-OFs and t-OFs. (f) The representative images of oil red O staining on n-OFs and t-OFs. (g) Relative oil red O-positive cell rate measured by ImageJ. ^*∗∗∗*^*P* <  0.001, ^*∗∗∗∗*^*P* <  0.0001vs n-OFs group by two-tailed Students' *t*-test.

PPAR-*γ*/C/EBP*α* signaling is important in adipogenic differentiation; as such, the expression of PPAR-*γ*/C/EBP*α* signaling related proteins as well as adipogenesis markers such as adiponectin and FABP-4 in n-OFs and t-OFs was determined using western blotting. The expressions of PPAR-*γ*, C/EBP*α*, adiponectin, and FABP-4 in t-OFs were significantly increased (Figures 2(a)–2(e)). Furthermore, oil red O staining revealed noticeable lipid accumulation in t-OFs, whereas no lipid accumulated in n-OFs (Figures 2(f), 2(g)). These data indicate that the number of red O-positive cells in t-OFs was nearly 3 times greater than that in n-OFs. Collectively, the findings demonstrate that t-OFs have a greater capacity for adipogenesis than n-OFs, which may be due to the high activation of PPAR-*γ*/C/EBP*α* signaling.

### 3.3. miR-96-5p Inhibition Reduces Adipogenesis of t-OFs

**Figure 3:**
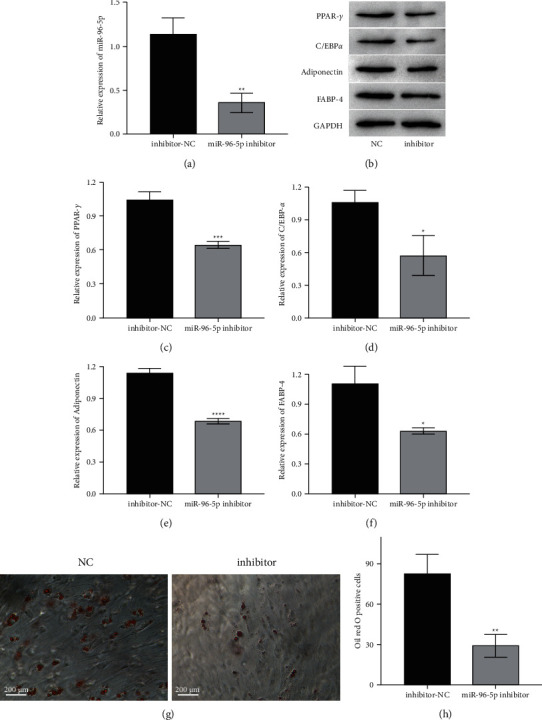
The role of miR-96-5p knockdown in adipogenic differentiation of t-OFs. (a) The relative expression of miR-96-5p in different groups of t-OFs. (b) The representative bands of western blotting for PPAR-*γ*, C/EBP*α*, adiponectin, and FABP-4. (c–f), The relative expression levels of PPAR-*γ*, C/EBP*α*, adiponectin, and FABP-4 in different groups of t-OFs. (g) The representative images of oil red O staining on t-OFs. (h) Relative oil red O-positive cell rate measured by ImageJ. ^*∗*^*P* <  0.05, ^*∗∗*^*P* <  0.01, ^*∗∗∗*^*P* <  0.001, ^*∗∗∗∗*^*P* <  0.0001, vs inhibitor-NC group by two-tailed Students' *t*-test.

To confirm the role of miR-96-5p in t-OF adipogenesis, a miR-96-5p inhibitor was transfected into t-OFs, and the expression of miR-96-5p and related proteins as well as t-OF lipid accumulation were measured. The miR-96-5p inhibitor significantly impeded the expression of miR-96-5p in t-OFs (Figure 3(a)). Also, the miR-96-5p inhibitor significantly decreased the expressions of PPAR-*γ*, C/EBP*α*, adiponectin, and FABP-4 in t-OFs (Figures 3(b)–3(f)). Furthermore, miR-96-5p inhibition reduced t-OF lipid accumulation (Figures 3(g), 3(h)); of note, following miR-96-5p inhibition, the positive rate of oil red O staining in t-OFs cells decreased by more than 1.5 times. These findings suggest that knocking out miR-96-5p can inhibit t-OF adipogenesis.

### 3.4. miR-96-5p Targets and Inhibits the Expression of Smad7

**Figure 4:**
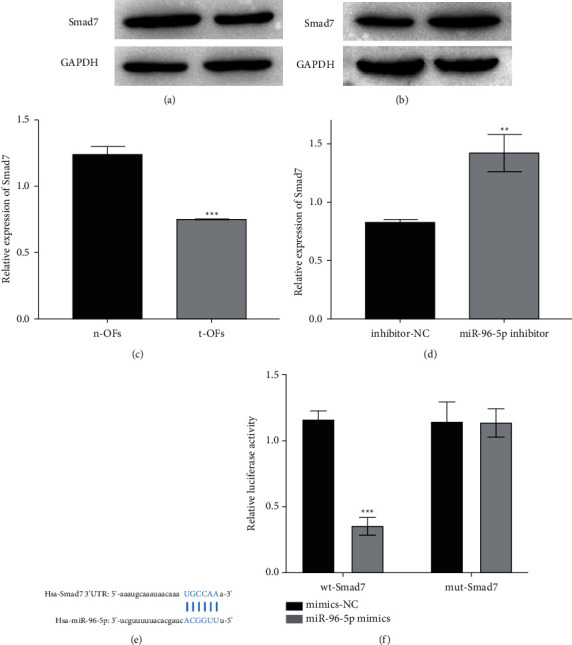
The relationship between miR-96-5p and Smad7. (a) The expression of Smad7 in n-OFs and t-OFs. (b) The expression of Smad7 in t-OFs with or without miR-96-5p inhibitor transfection. (c) The binding site between miR-69-5p and 3'UTR of Smad7. (d) The results of the dual-luciferase report assay. ^*∗∗*^*P* <  0.01, ^*∗∗∗*^*P* <  0.001, vs their control group, (a, b) were detected by two-tailed Students' *t*-test, and (c) was detected by two-way ANOVA.

Previous research revealed that Smad7 is involved in the regulation of adipogenesis [29, 30]; as such, we examined the expression of Smad7 in t-OFs western blotting. The results showed lower Smad7 expression in t-OFs than in n-OFs (Figure 4(a)). Meanwhile, miR-96-5p inhibition promoted Smad7 expression in t-OFs (Figure 4(b)). Furthermore, Smad7 was predicted to be one of the target genes of miR-96-5p, and there was a binding site between miR-96-5p and 3'UTR of Smad7 (Figure 4(c)). A dual-luciferase reporter assay confirmed that miR-96-5p could precisely bind to wild-type Smad7 3'UTR (Figure 4(d)). These findings suggested that miR-96-5p may promote t-OF adipogenesis by targeting Smad7.

### 3.5. miR-96-5p Inhibition Decreases t-OF Adipogenesis by Increasing Smad7 Expression

**Figure 5:**
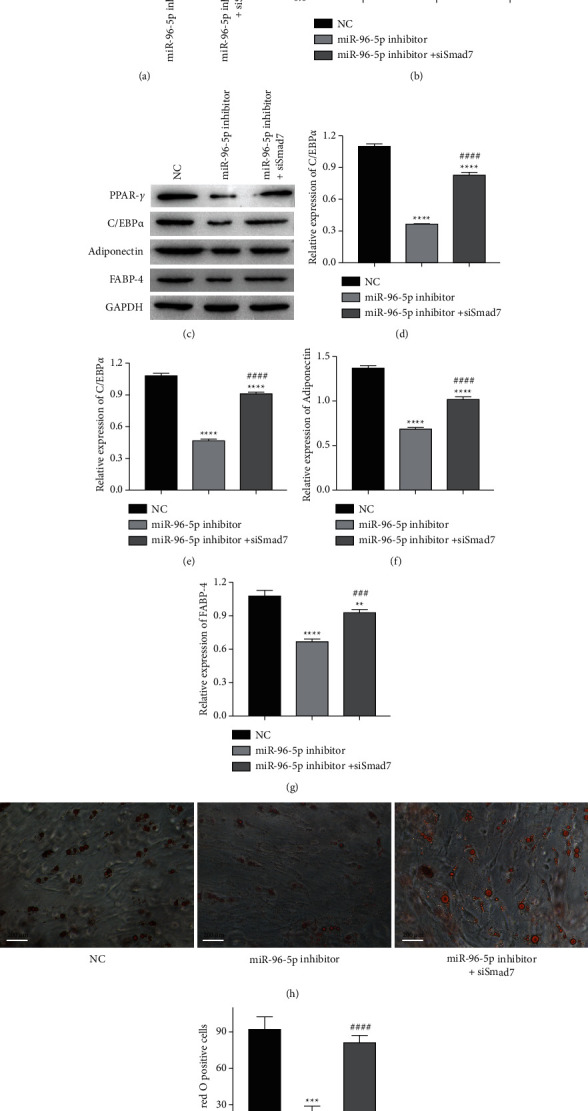
The effect of Smad7 on miR-96-5p knockdown mediated the inhibition of t-OFs adipogenesis. (a, b) The expression of Smad7 in each group of t-OFs. (c–g) The expression of PPAR-*γ*, C/EBP*α*, adiponectin, and FABP-4 in each group of t-OFs. (h, i) The representative images of oil red O staining on t-OFs and relative oil red O-positive cell rate. ^*∗∗*^*P* <  0.01, ^*∗∗∗*^*P* <  0.001, ^*∗∗∗∗*^*P* <  0.0001 vs NC group, ^##^*P* <  0.01, ^###^*P* <  0.001, ^####^*P* <  0.0001, vs miR-96-5p inhibitor group by one-way ANOVA.

To investigate whether miR-96-5p promotes t-OFs adipogenic differentiation of t-OFs, miR-96-5p inhibitor and siSmad7 were cotransfected into t-OFs, and protein expressions and lipid accumulation in t-OFs were measured. Smad7 expression was increased by inhibiting miR-96-5p but decreased by siSmad7 (Figures 5(a) and 5(b)). siSmad7 also increased the expressions of PPAR-*γ*, C/EBP*α*, adiponectin, aind FABP-4 in t-OFs, which were inhibited by the miR-96-5p inhibitor (Figures 5(c)–5(g)). Of note, the lipid accumulation in t-OFs inhibited by miR-96-5p inhibition was also reversed by siSmad7 (Figures 5(h) and 5(i)), and after miR-96-5p inhibition, the positive rate of oil red O staining in t-OFs cells decreased by about 2 times, but after adding siSmad7 to inhibit the expression of Smad7, the positive rate of oil red O staining in t-OFs cells increased to about 90% of that in the control group. These findings suggested that miR-96-5p promotes t-OF adipogenic differentiation by specifically inhibiting Smad7 expression and that miR-96-5p inhibition can block t-OF adipogenic differentiation by upregulating Smad7 expression and inhibiting PPAR-*γ*/C/EBP*α* signaling.

## 4. Discussion

Thyroid-related ophthalmopathy (TAO) is a common ophthalmic disease, with the highest incidence among orbital diseases that can cause blindness [1, 2]. TAO is distinguished by an increase in adipose/connective tissues. Compelling evidence shows that TAO symptoms are caused by inflammation of the orbital connective tissue [11, 12]. Also, researchers have demonstrated that adipogenesis mediated by adipogenic differentiation of orbital fibroblasts (OFs) plays an important role in TAO progression [16, 17]. Previous research has shown that many genes and signal transduction pathways, such as insulin-like growth factor-1 receptor, FABP4/5, APOE, PPARG and ADIPOQ, PI3K Akt signal transduction, cAMP signal transduction, AGE-RAGE signal, and Wnt signal pathway, are involved in the adipogenesis of TAO patients [40, 41]. This study confirmed that OFs isolated from adipose tissues of TAO patients had greater adipogenic differentiation ability than OFs isolated from adipose tissues of non-TAO participants. PPAR-*γ*/C/EBP*α* signaling is linked to adipogenic differentiation; adiponectin and FABP-4 are adipogenic markers, and their expressions were upregulated in t-OFs.

Recent evidence shows that miRNAs play an important role in the development of TAO. For instance, Jang et al. revealed that miR-27 could inhibit the adipogenic differentiation of OFs in Graves' disease patients [16]. Elsewhere, miR-183 and miR-96 were found to potentially contribute to the progression of Graves' orbitopathy by regulating T cell activation [42], whereas miR-146a could regulate the fibrosis of OFs in Graves' orbitopathy [35, 43]. miR-96-5p was found to be significantly positively correlated with TAO severity [22], and it was also revealed to play a role in adipogenic differentiation of C2C12 myoblasts [21]. In the present study, we discovered that miR-96-5p was overexpressed in orbital tissues and OFs of TAO patients. Further research revealed that miR-96-5p could aggravate PPAR-*γ*/C/EBP*α* pathway-induced adipogenic differentiation of t-OFs by specifically inhibiting Smad7 expression. Inhibiting miR-96-5p, on the other hand, could inhibit t-OF adipogenic differentiation-mediated adipogenesis via Smad7/PPAR-*γ*/C/EBP*α*. Notably, in addition to adipogenic differentiation, OFs-mediated orbital fibrosis and inflammatory factor release are important in the development and progression of TAO [15]. Meanwhile, miRNA expression acted as a key regulator in OFs-mediated orbital fibrosis and inflammatory factor release. Previous research found that miR-146a could inhibit TGF-*β*-induced OFs fibrosis [35]. Other studies have demonstrated that miR-146a could promote OFs proliferation and proinflammatory IL-6 expression by targeting Notch2 [43, 44] and that miR-21 and miR-155 could also be involved in fibrosis and inflammation caused by OFs in orbital tissues [36, 45, 46]. These findings suggest that miRNAs play an important role in the development of TAO and that miRNAs may be potential biomarkers and therapeutic targets for TAO.

In conclusion, the present study demonstrates a role for miR-96-5p in adipogenesis of orbital fibroblast in TAO patients. The results revealed that miR-96-5p is highly expressed in orbital tissues of TAO patients and t-OFs and that miR-96-5p can promote t-OF adipogenesis by specifically inhibiting Smad7 expression. Furthermore, miR-96-5p knockdown potentially inhibits t-OF adipogenic differentiation via Smad7/PPAR-*γ*/C/EBP*α* signaling. This research suggests that miR-96-5p could be a biomarker and potential therapeutic target for TAO. However, because this is our preliminary work to investigate the role of miR-96-5p in TAO, it has significant limitations. We will improve the experimental design in subsequent studies, including cell experiments, animal model construction, and clinical sample detection and verification.

## Data Availability

The datasets used and/or analyzed during the current study are available from the corresponding author upon reasonable request.

## References

[1] Le Moli R., Malandrino P., Russo M. (2020). Corticosteroid pulse therapy for Graves’ ophthalmopathy reduces the relapse rate of Graves’ hyperthyroidism. *Frontiers in Endocrinology*.

[2] Vagge A., Federico B., Chiara D. N. (2020). In vivo confocal microscopy morphometric analysis of meibomian glands in patients with graves ophthalmopathy. *Cornea*.

[3] Fox T. J., Anastasopoulou C. (2020). Graves orbitopathy. *StatPearls.*.

[4] Antonelli A., Fallahi P., Elia G. (2020). Graves’ disease: clinical manifestations, immune pathogenesis (cytokines and chemokines) and therapy. *Best Practice & Research Clinical Endocrinology & Metabolism*.

[5] Taylor P. N., Zhang L., Lee R. W. J. (2020). New insights into the pathogenesis and nonsurgical management of Graves orbitopathy. *Nature Reviews Endocrinology*.

[6] Wu S., Cai T., Chen F., He X., Cui Z. (2015). Genetic associations of FCRL3 polymorphisms with the susceptibility of Graves ophthalmopathy in a Chinese population. *International Journal of Clinical and Experimental Medicine*.

[7] Jurecka-Lubieniecka B., Ploski R., Kula D. (2014). Association between polymorphisms in the TSHR gene and Graves’ orbitopathy. *PloS one*.

[8] Planck T., Shahida B., Parikh H. (2014). Smoking induces overexpression of immediate early genes in active Graves’ ophthalmopathy. *Thyroid*.

[9] Ferrari S. M., Fallahi P., Antonelli A., Benvenga S. (2017). Environmental issues in thyroid diseases. *Frontiers in Endocrinology*.

[10] Huang Y., Fang S., Zhang S., Zhou H. (2020). Progress in the pathogenesis of thyroid-associated ophthalmopathy and new drug development. *Taiwan journal of ophthalmology*.

[11] Li H., Ma C., Liu W., He J., Li K. (2020). Gypenosides protect orbital fibroblasts in Graves ophthalmopathy via anti-inflammation and anti-fibrosis effects. *Investigative Opthalmology & Visual Science*.

[12] Slentz D. H., Nelson C. C., Smith T. J. (2020). Teprotumumab: a novel therapeutic monoclonal antibody for thyroid-associated ophthalmopathy. *Expert Opinion on Investigational Drugs*.

[13] Łacheta D., Piotr M., Alicja G. (2019). Immunological aspects of graves’ ophthalmopathy. *BioMed research international*.

[14] Wang Z.-M., Wang Z., Lu Y. (2019). The role of cell mediated immunopathogenesis in thyroid-associated ophthalmopathy. *International Journal of Ophthalmology*.

[15] Dik W. A., Virakul S., van Steensel L. (2016). Current perspectives on the role of orbital fibroblasts in the pathogenesis of Graves’ ophthalmopathy. *Experimental Eye Research*.

[16] Jang S. Y., Chae M. K., Lee J. H., Lee E. J., Yoon J. S. (2019). MicroRNA-27 inhibits adipogenic differentiation in orbital fibroblasts from patients with Graves’ orbitopathy. *PLoS one*.

[17] Kim J. Y., Park S., Lee H.-J., Lew H., Kim G. J. (2020). Functionally enhanced placenta-derived mesenchymal stem cells inhibit adipogenesis in orbital fibroblasts with Graves’ ophthalmopathy. *Stem Cell Research & Therapy*.

[18] Peyster R., Ginsberg F., Silber J., Adler L. (1986). Exophthalmos caused by excessive fat: CT volumetric analysis and differential diagnosis. *American Journal of Roentgenology*.

[19] Takuse Y., Watanabe M., Inoue N. (2017). Association of IL-10-regulating MicroRNAs in peripheral blood mononuclear cells with the pathogenesis of autoimmune thyroid disease. *Immunological Investigations*.

[20] Qin L., Chen Y., Niu Y. (2010). A deep investigation into the adipogenesis mechanism: profile of microRNAs regulating adipogenesis by modulating the canonical Wnt/*β*-catenin signaling pathway. *BMC Genomics*.

[21] Hou Y., Fu L., Li J. (2018). Transcriptome analysis of potential miRNA involved in adipogenic differentiation of C2C12 myoblasts. *Lipids*.

[22] Martínez-Hernández R., Miguel S.-N., Ana S.-S. (2018). A MicroRNA signature for evaluation of risk and severity of autoimmune thyroid diseases. *Journal of Clinical Endocrinology & Metabolism*.

[23] Liu Z., Cui Y., Wang S. (2021). MiR-96-5p is an oncogene in lung adenocarcinoma and facilitates tumor progression through ARHGAP6 downregulation. *Journal of Applied Genetics*.

[24] Wang T., Xu Y., Liu X., Zeng Y., Liu L. (2021). miR-96-5p is the tumor suppressor in osteosarcoma via targeting SYK. *Biochemical and biophysical research communications*.

[25] Li R., Chen Y., Wu J. (2021). LncRNA FGF14-AS2 represses growth of prostate carcinoma cells via modulating miR-96-5p/AJAP1 axis. *Journal of Clinical Laboratory Analysis*.

[26] Vallelunga A., Iannitti T., Capece S. (2021). Serum miR-96-5P and miR-339-5P are potential biomarkers for multiple system Atrophy and Parkinson’s disease. *Frontiers in Aging Neuroscience*.

[27] Wang Q. L., Li H. F., Wang D. P. (2019). Effect of GGCX on the differentiation function of osteoporosis bone marrow mesenchymal stem cells through regulating TGF*β*/smad signaling pathway. *European Review for Medical and Pharmacological Sciences*.

[28] Li N., Lee W. Y.-W., Lin S.-E. (2014). Partial loss of Smad7 function impairs bone remodeling, osteogenesis and enhances osteoclastogenesis in mice. *Bone*.

[29] Kim B.-H., Han S., Lee H. (2015). Metformin enhances the anti-adipogenic effects of atorvastatin via modulation of STAT3 and TGF-*β*/Smad3 signaling. *Biochemical and biophysical research communications*.

[30] Ouyang D., Xu L., Zhang L. (2016). MiR-181a-5p regulates 3T3-L1 cell adipogenesis by targetingSmad7andTcf7l2. *Acta biochimica et biophysica Sinica*.

[31] Li Y., Jin D., Xie W. (2018). PPAR-*γ* and Wnt regulate the differentiation of MSCs into adipocytes and osteoblasts respectively. *Current Stem Cell Research and Therapy*.

[32] Zhuang H., Zhang X., Zhu C. (2016). Molecular mechanisms of PPAR-*γ*; governing MSC osteogenic and adipogenic differentiation. *Current Stem Cell Research and Therapy*.

[33] Vallée A., Lecarpentier Y., Guillevin R., Vallée J. N. (2017). Interactions between TGF-*β*1, canonical WNT/*β*-catenin pathway and PPAR *γ* in radiation-induced fibrosis. *Oncotarget*.

[34] Hao M., Sun J., Zhang Y. (2020). Exploring the role of SRC in extraocular muscle fibrosis of the Graves’ ophthalmopathy. *Frontiers in Bioengineering and Biotechnology*.

[35] Jang S. Y., Park S. J., Chae M. K., Lee J. H., Lee E. J., Yoon J. S. (2018). Role of microRNA-146a in regulation of fibrosis in orbital fibroblasts from patients with Graves’ orbitopathy. *British Journal of Ophthalmology*.

[36] Tong B. D., Xiao M. Y., Zeng J. X., Xiong W. (2015). MiRNA-21 promotes fibrosis in orbital fibroblasts from thyroid-associated ophthalmopathy. *Molecular Vision*.

[37] Yoon J. S., Chae M. K., Jang S. Y., Lee S. Y., Lee E. J. (2012). Antifibrotic effects of quercetin in primary orbital fibroblasts and orbital fat tissue cultures of Graves’ orbitopathy. *Investigative Opthalmology & Visual Science*.

[38] Yuan R., Dai X., Li Y., Li C., Liu L. (2021). Exosomes from miR-29a-modified adipose-derived mesenchymal stem cells reduce excessive scar formation by inhibiting TGF-*β*2/Smad3 signaling. *Molecular Medicine Reports*.

[39] Livak K. J., Schmittgen T. D. (2001). Analysis of relative gene expression data using real-time quantitative PCR and the 2−ΔΔCT method. *Methods*.

[40] Kim D. W., Taneja K., Hoang T. (2021). Transcriptomic profiling of control and thyroid-associated orbitopathy (TAO) orbital fat and TAO orbital fibroblasts undergoing adipogenesis. *Investigative Opthalmology & Visual Science*.

[41] Jung S., Choi Y. J., Park T. K. (2021). Wnt signalling inhibits adipogenesis in orbital fibroblasts from patients with Graves’ orbitopathy. *British Journal of Ophthalmology*.

[42] Thiel J., Alter C., Luppus S. (2019). MicroRNA-183 and microRNA-96 are associated with autoimmune responses by regulating T cell activation. *Journal of Autoimmunity*.

[43] Jang S. Y., Chae M. K., Lee J. H., Lee E. J., Yoon J. S. (2016). Role of miR-146a in the regulation of inflammation in an in vitro model of Graves’ orbitopathy. *Investigative Opthalmology & Visual Science*.

[44] Wang N., Chen F.-E., Long Z.-W. (2017). Mechanism of MicroRNA-146a/notch2 signaling regulating IL-6 in Graves ophthalmopathy. *Cellular Physiology and Biochemistry*.

[45] Lee J.-Y., Yun M., Paik J.-S., Lee S.-B., Yang S.-W. (2016). PDGF-BB enhances the proliferation of cells in human orbital fibroblasts by suppressing PDCD4 expression via up-regulation of microRNA-21. *Investigative Opthalmology & Visual Science*.

[46] Li K., Du Y., Jiang B. L., He J. F. (2014). Increased microRNA-155 and decreased microRNA-146a may promote ocular inflammation and proliferation in Graves’ ophthalmopathy. *Medical Science Monitor: International Medical Journal of Experimental and Clinical Research*.

